# SOFIA^®^RSV: prospective laboratory evaluation and implementation of a rapid diagnostic test in a pediatric emergency ward

**DOI:** 10.1186/s12879-017-2557-8

**Published:** 2017-06-26

**Authors:** Léa C. Tran, Céline Tournus, Julia Dina, Rémy Morello, Jacques Brouard, Astrid Vabret

**Affiliations:** 1 Normandy University, UNICAEN, EA 4655-U2RM, EA 2656-GRAM, Caen, France; 2 Department of Virology, National Reference Laboratory for Measles and Paramyxoviridae, CHU de Caen, Normandy, Caen, France; 3 Department of Pediatrics, CHU de Caen, Normandy, Caen, France; 4 Department of Statistics and Clinical Research, CHU de Caen, Normandy, Caen, France

**Keywords:** Respiratory syncytial virus, Rapid diagnostic tests, SOFIA^®^RSV, Point-of-care testing, Bronchiolitis, Respiratory infection, Pediatric emergency ward

## Abstract

**Background:**

Respiratory syncytial virus (RSV) is responsible for severe respiratory infections and higher costs in medical care. The two aims of this work were to assess the performances of SOFIA^®^RSV tests in “real-life-laboratory” conditions (study 1) and implemented at point-of-care testing in a pediatric emergency department (ED, study 2), during two consecutive winter seasons.

**Methods:**

In study 1, fresh nasopharyngeal swabs from patients of all ages were sampled in 1.5 ml of Universal virological Transport Medium (UTM) and prospectively tested using SOFIA^®^RSV tests. In study 2, conducted in a pediatric ED, nasopharyngeal swabs were placed in 3 ml of UTM. All SOFIA^®^RSV tests were confirmed by molecular testing, considered as reference method. The epidemiological and clinical features of tested patients, as well as the care of these patients after obtaining quick results were evaluated.

**Results:**

The sensitivities of SOFIA^®^RSV in infants (aged under 24 months) performed in the laboratory and in the pediatric ED were respectively 95% (95% CI: 86.8–98.1) and 74.8% (95% CI: 68.0–80.9) compared to PCR. In study 1, the sensitivity among children (from 2 to 15 years old) and adults (above 15 years old) dropped to 45% (95% CI: 23.1–68.5) and 59% (95% CI: 32.9–81.6), respectively. In study 2, there were some differences in bed-management of SOFIA^®^RSV positive compared to SOFIA^®^RSV negative infants.

**Conclusions:**

SOFIA^®^RSV tests performed in the laboratory and in the pediatric ED show high and satisfactory sensitivities among young children under 24 months, which supports its robustness and reliability. However, the impact of these tests on patient care at point-of-care cannot be clearly assessed when considering the limits of the study 2 design.

**Electronic supplementary material:**

The online version of this article (doi:10.1186/s12879-017-2557-8) contains supplementary material, which is available to authorized users.

## Background

Respiratory syncytial virus (RSV) is a common ubiquitous pathogen responsible for mild upper respiratory tract infection in most children and healthy adults. In infants, RSV is mainly associated with acute lower respiratory infections (ALRI, which includes bronchiolitis and pneumonia) during the winter months in temperate regions of the northern hemisphere. In elderly and immunocompromised patients, RSV has been known to cause severe respiratory failure, extended hospitalizations, higher mortality, with symptoms similar to those associated with seasonal influenza [1, 2]. If 98% of all infants have been infected by RSV at least once by the age of two [3], primary infection does not protect against reinfection but likely lessens the severity of later infections. In infants and young children, RSV is more likely to move into the lower respiratory tract, and infect ciliated followed by non-ciliated cells [4].

RSV is a major public health problem worldwide, since its circulation generates a significant excess activity in pediatric departments, including pediatric emergency departments (ED). In 2005, 3.4 million young children were hospitalized worldwide for severe ALRI associated with RSV [5]. In a 5-year prospective data analysis, the RSV hospitalization rate was 5.2 per 1000 infants, among whom infants under 1 month of age were at highest risk of hospitalization [6]. A previous epidemiologic study conducted in 2009 at Caen University Hospital in infants with respiratory symptoms during winter season had already highlighted significant associations between RSV infection, higher risk of hospitalization and higher clinical severity scores [7]. In a meta-analysis including 82,000 patients who had been infected by RSV before 3 years old (y/o), Regnier et al. [8] found that 21.9% of children hospitalized for an RSV infection during their first year of life develop asthma before 5 y/o. However, diagnosing RSV infection based only on respiratory symptoms is not possible since the symptoms most often found in RSV infections are nonspecific, requiring virological confirmation [3].

Rapid diagnostic tests (RDT) for RSV are frequently used in emergency departments to guide and establish the most accurate and quickest RSV diagnosis possible, despite their insufficient sensitivity [9, 10]. A second generation, automated RDT, SOFIA Fluorescent Immunoassay for RSV (SOFIA^®^RSV, QUIDEL, San Diego, CA), was introduced in France in 2013. This assay allowed for quick RSV detection and enabled better decision-making in bed-management, thus enhancing infection control. These newer standardized tests are an improvement over the performances of classical manual RDT [11]. Indeed, they include a cell lysis phase, a time-controlled incubation phase inside the machine as well as an objective automated lecture. Their use at point-of-care testing (POCT) should enhance patient care, which begins in the pediatric ED.

This report combines two studies, study 1 and study 2, each having its own objective. The objective of study 1 was to assess the analytical performances of SOFIA^®^RSV tests under “real-life laboratory” conditions, including samples from patients of all ages. This prospective study was conducted in 2013. The objective of study 2 was to assess the performance of the SOFIA^®^RSV test implemented at POCT in a pediatric ED during four winter months (from November 2013 to March 2014).

## Methods

### Samples and patients

Study 1 focused on fresh nasopharyngeal swabs sent to the virology laboratory of Caen University Hospital, France, for virological diagnosis. Eight nasopharyngeal swabs received in laboratory in 1.5 mL of Universal virological Transport Medium (UTM) were prospectively included every working day (five days a week), over a 10 weeks period from December 3, 2012 (week 49) to February 11, 2013 (week 6). The selection criteria were based on the age of sampled patients. Among these 8 nasopharyngeal swabs, 4 samples were provided from patients under 2 y/o, 2 from patients aged from 2 to 15 y/o, and 2 from patients over 15 y/o. Consequently, we defined 3 age groups: Infant group (under 2 y/o), Child group (from 2 to 15 y/o), and Adult group (over 15 y/o). Overall, 401 samples were provided for the study. Caen University Hospital serves the Calvados region, with a general population of 689,439 (data from l’Institut national de la statistique et des études économiques [Insee], January 1, 2013). During the study period, there were 20,332 admissions, with a mean of 290 admissions per day.

Study 2 was conducted over a 20 weeks winter period from November 13, 2013 (week 46) to March 31, 2014 (week 13) for POCT, in the pediatric ED of Caen University Hospital. This unit serves the Calvados region with 124,920 children aged under 15 y/o (data from l’Insee, on January 1, 2014). During the study 2 period, there were 8222 admissions, with a mean of 59 admissions per day (including a mean hospitalization rate of 21%). A test was prescribed when the patient displayed symptoms of ALRI. All children under 15 y/o who had a SOFIA^®^RSV test and for whom parental or guardian consent for exams, treatments and/ or hospitalization was gained were included. For each patient, a standardized form was filled. Data were retrospectively extracted from each patient’s medical records in the ED, collated on Microsoft Excel sheet and analyzed. We collected data that were present in all patient’s medical records, including clinical features (age, gestational age, presence or absence of fever ≥38 °C), final diagnosis, chest radiography (yes/no), prescribed treatments (antibiotics, corticosteroids, aerosols), the decision of hospitalization in different units (observation unit, pediatric intensive care unit [PICU], and general pediatrics ward) and length of stay in the general pediatrics ward. At the end of study 2, a satisfaction survey was sent to 13 pediatric consultants working in the ED, including four questions and one space for comments (Additional file 1).

### Viral detection

In study 1, all the respiratory samples were tested using SOFIA^®^RSV, Direct Immunofluorescence Assay, detecting eight viral targets (direct fluorescent antibody, DFA): influenza virus type A, B (FluA, FluB), human RSV (hRSV), human metapneumovirus (hMPV), human adenovirus (hAdV), and human parainfluenza viruses 1, 2, 3 (hPIVs). All study samples were inoculated in cell culture (MRC5 cells) and were then tested using molecular tests: Respifinder Smart22 Fast^®^, Eurogentec if DFA tested negative, and RSV/hMPV r-gene^®^ Biomerieux, if DFA tested positive for RSV. These two PCR tests showed equivalent sensitivity for RSV detection and were considered as a RSV detection reference method [12].

In study 2, two SOFIA^®^RSV analyzers were set up in the pediatric ED of Caen University Hospital. Nurses had been previously authorized by virologists to perform the rapid diagnostic tests on fresh respiratory specimens, using the modified standardized protocol: these tests were designed to be performed from a respiratory specimen sampled in 1 mL of UTM. After a sample extraction, an aliquot was pipetted onto the test cassette. Incubation (15 min) and reading (1 min) phases were performed as per mandatory protocol within the SOFIA^®^RSV analyzer. Rapid positive and negative results were provided after 15 min. In our study, nasopharyngeal swab samples were placed in 3 mL of UTM (compared to 1 mL according to the manufacturer’s protocol) and were systematically sent to the laboratory. This volume of 3 ml is necessary to carry out PCR tests for other respiratory viruses and also to prepare the storage of aliquots. All positive tests were confirmed by specific RT-PCR targeted RSV (molecular tests are considered as reference testing). For these SOFIA^®^RSV positive tests, viral codetection was not examined. All the negative samples were tested with a multiplex PCR (Respifinder^®^ Smart 22 fast, Pathofinder), capable of detecting 21 respiratory pathogens, including 17 viruses: Flu A, B, Flu A H_1_N_1v_, hRSV A,B, hMPV, human rhinovirus/enterovirus (hRV/EV), PIV 1, 2, 3, 4, hADV, Bocavirus, and human Coronaviruses (hCoV) NL63, HKU1, 229E, OC43.

### Statistical analysis

The sensitivities, specificities, the positive and negative predictive values, and the positive and negative likelihood ratios were calculated with confidence intervals of 95%, using PCR as the gold standard. In study 2, for the 19 SOFIA^®^RSV samples tested in the pediatric ED which have been included (all SOFIA^®^RSV positive), confirmation by RT-PCR has not been done for technical reasons and have been excluded in the calculation of SOFIA^®^RSV performances. Chi^2^ and Fisher tests were used for statistical comparisons. The type I error was 5%.

## Results

### Study 1: Prospective evaluation of SOFIA^®^RSV in a “real-life laboratory”

What ‘real-life’ means is that each sample has been managed as per routine protocol. This situation has the advantage of allowing the testing of samples in conditions closest to those of a laboratory of medical virology (including standard operating procedures) as opposed to a situation where the analysis are performed by a technician dedicated to the study and only that study, not taking into account the daily laboratory work load of any hospital routine laboratory.

Study 1 included 401 nasopharyngeal swabs: 49.6% were sampled from the Infant group (*n* = 199), 24.9% from the Child group (*n* = 100), and 25.4% from the Adult group (*n* = 102). The Adult group (above 15 years-old) was divided into patients aged between 15 and 65 y/o (35.3%, *n* = 40) and patients above 65 y/o (64.7%, *n* = 62).

**Fig. 1:**
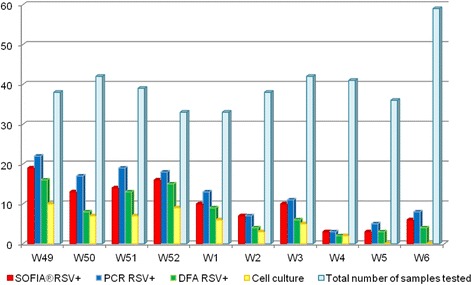
Study 1. Distribution of the respiratory samples by week 49 (December 3, 2012) to week 6 (February 11, 2013). The total of 101 SOFIA^®^RSV positive samples are in *red*, the 123 RSV PCR samples positive are in *blue*, the 80 RSV DFA positive samples are in *green* and the 53 cell culture positive samples are in *yellow*. The 401 respiratory samples of study 1 are in *light-blue*

Among these 401 samples, 123 (30.7%) tested RSV positive using the molecular method, 101 (25.2%) using SOFIA^®^RSV, 80 (19.9%) using RSV DFA, and 53 (13.2%) using cell culture (Fig. 1). The sensitivities, specificities, positive, and negative predicted values (PPV and NPV) are detailed in Table 1. Among the 123 PCR RSV positive samples, SOFIA^®^RSV displayed an overall sensitivity of 78.8% (95% in the Infant group, 45% in the Child group and 59%, and in the Adult group) compared to PCR. RSV DFA displayed an overall sensitivity of 63.4% (81% in the Infant group, 25% in the Child group and 23.5% in the Adult group) compared to PCR. Both SOFIA^®^RSV and DFA showed high specificities of 98.5% and 99.6%, respectively, compared to PCR. Negative predicted values of SOFIA^®^RSV and RSV DFA were 91.3% (96.6% in the Infant group, 88% in the Child group and 92% in the Adult group), and 86% (87.6% in the Infant group, 84% in the Child group and 86.7% in the Adult group), respectively.

**Table 1:** Study 1. The performance characteristics of SOFIA^®^RSV, RSV DFA, and cell culture (MRC5)

	Sensitivity (%) (95% CI)			Specificity (%) (95% CI)			Positive predictive value (%) (95% CI)			Negative predictive value (%)		
	SOFIA RSV	DFA RSV	MRC5 RSV	SOFIA RSV	DFA RSV	MRC5 RSV	SOFIA RSV	DFA RSV	MRC5 RSV	SOFIA RSV	DFA RSV	MRC5 RSV
Total (*n* = 401)	78.8 (70.6–85.7)	63.4 (54.2–71.9)	42.2 (33.4–51.5)	98.5 (96.4–99.6)	99.6 (98.0–100.0)	99.6 (98.0–100.0)	96.0 (90.2–98.9)	98.7 (93.1–100.0)	98.0 (89.9–100.0)	91.3 (87.6–94.3)	86.0 (81.7–89.6)	79.6 (75.0–83.7)
Infant group (*n* = 199)	95.0 (86.8–98.1)	81.0 (71.2–88.8)	54.0 (43.0–65.0)	99.0 (95.2–100.0)	100.0 (97.4–100.0)	100.0 (95.2–100.0)	99.0 (93.3–100.0)	100.0 (95.7–100.0)	98.0 (88.7–100.0)	96.6 (90.4–98.6)	87.6 (80.8–92.8)	74.3 (66.6–81.1)
Children group (*n* = 100)	45.0 (23.1–68.5)	25.0 (8.7–49.1)	30.0 (11.9–54.3)	97.5 (91.3–99.7)	98.7 (93.2–100.0)	98.7 (96.3–100.0)	82.0 (48.2–97.7)	83.3 (35.9–99.6)	100.0 (60.7–100.0)	88.0 (79.0–93.7)	84.0 (75.0–90.8)	85.0 (76.3–91.6)
Adult group (*n* = 102)	59.0 (32.9–81.6)	23.5 (6.8–49.9)	0.0 (0.0–16.2)	100.0 (96.5–100.0)	100.0 (96.5–100.0)	100.0 (96.5–100.0)	100.0 (74.1–100.0)	100.0 (47.3–100.0)	/	92.0 (84.9–96.9)	86.7 (78.4–92.7)	83.0 (74.7–90.0)

Sensitivities, specificities, positive and negative predicted values (PPV and NPV) of SOFIA^®^RSV, RSV DFA, and cell culture (MRC5) calculated on the 401 respiratory samples of study 1. The PCR test was used as the reference test

### Study 2: Evaluation of SOFIA^®^RSV at POCT in a pediatric ED

**Fig. 2:**
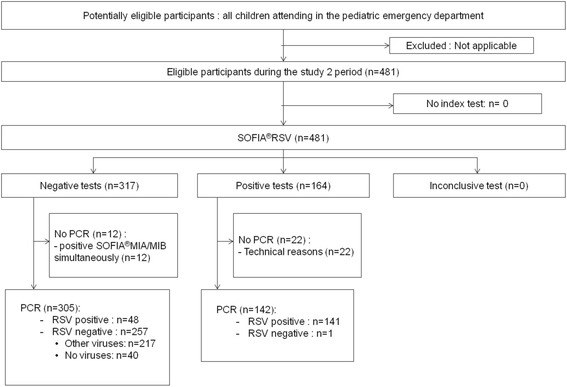
Study 2. Flow-chart of participants (November 13, 2013 to March 31, 2014). Eligible criteria: all children under 15 years-old who had a SOFIA^®^RSV test in the pediatric emergency department (parental or guardian consents gained)

The aim of study 2 was to describe the implementation of SOFIA^®^RSV in a pediatric ED in order to assess its performance at POCT, particularly among infants, whom group presented the highest values of sensitivity in study 1 (Fig. 2).

481 SOFIA^®^RSV tests were performed on 432 patients (sex ratio M/F: 1.3). Some patients presented for more than once and had at least two tests. For 20 weeks, there were an average of 24 tests per week and 6.75 tests per weekend. Four hundred and fifty-seven tests (95%) were performed on patients under 2 y/o: Of these, 263 (57.5%) were infants under 3 months of age, 90 (19.7%) were aged between 4 and 6 months and 104 (22.8%) were aged between 7 and 24 months. Since the vast majority in this population was composed of infants aged under 6 months, we chose to focus the statistical analysis on infants (aged under 24 months).

**Fig. 3:**
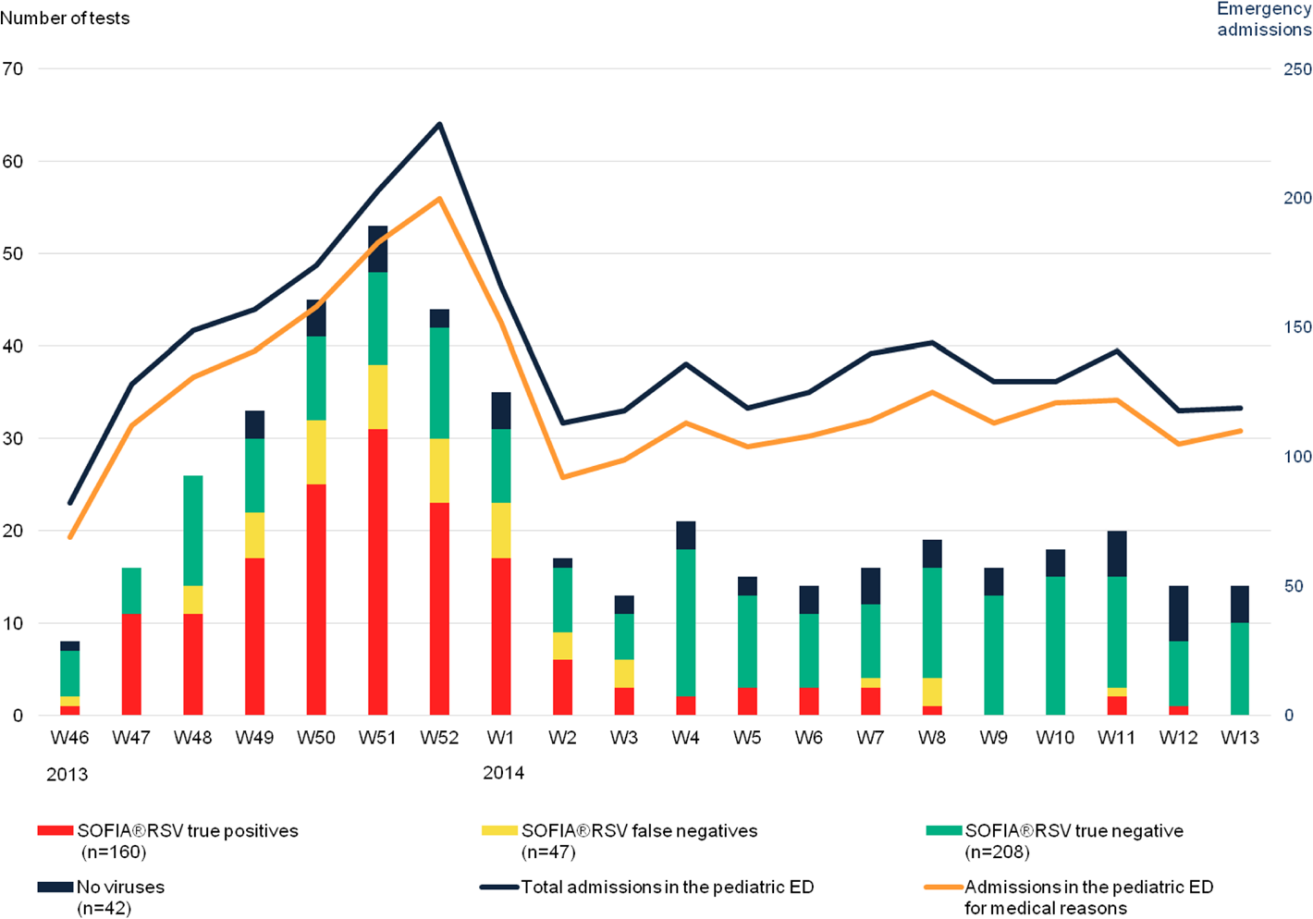
Study 2. Weekly distribution of nasopharyngeal swabs and their virological outcomes in infants (≤2 y/o) in the pediatric emergency department. Different colors correspond to different results of virological tests. *Dark blue* and *green squares* correspond to SOFIA^®^RSV negative (Group 1), with no viruses detected by PCR (*dark blue*) and PCR positive for other viruses than RSV (*green*), *yellow squares* correspond to SOFIA^®^RSV negative and PCR positive for RSV (Group 2) and *red squares* correspond to SOFIA^®^RSV positive (Group 3). The *blue curve* represents the total number of infants admitted in the emergency department (for medical and surgical emergencies) and the *orange curve* corresponds to the number of infants admitted for medical reasons

The weekly distribution of SOFIA^®^RSV tests among infants is described by Fig. 3. The largest number of SOFIA^®^RSV tests (*n* = 53) was performed in week 51 (from 16th to 22th of December 2013) and the largest number of ED visits (*n* = 200) was observed in week 51 (from the 23rd to 29th of December 2013). Of the 2819 visits, 2472 (87.7%) came for medical reasons and the 347 (12.3%) others came for surgical reasons. Overall, in infants, 87% of samples analyzed were positive for at least one respiratory virus. This positive result was as high as 100% at weeks 47 and 48.

The performance characteristics of SOFIA^®^RSV in infants, at POCT and compared to PCR, were: sensitivity 74.8%, specificity 99.6%, positive predictive value 99.3%, and negative predictive value 84.2%. Positive and negative likehood ratio were respectively 187 and 0.253 (Table 2). Among the 457 tested infants, 160 (35%) were SOFIA^®^RSV positive; 47 (10.3%) were SOFIA^®^RSV negative and RSV PCR positive; 208 (45.5%) were SOFIA^®^RSV negative and PCR positive for viruses other than RSV; and 42 (9.2%) were SOFIA^®^RSV negative, with no virus detected by PCR. Among the 47 SOFIA^®^RSV negative and RSV PCR positive, one or two other viruses were detected in 24 and 3 samples, respectively.

**Table 2:** Study 2. SOFIA^®^RSV performance characteristics compared to PCR in infants at point-of-care

	SOFIA RSV +	SOFIA RSV-	Total	SOFIA^®^RSV test performances compared to PCR (95% CI)
PCR RSV +	140	47	187	Sensitivity: 74.8% (68.0–80.9)
				Specificity: 99.6% (97.8–100.0)
PCR RSV -	1	250	251	PPV: 99.3% (96.1–100.0)
				NPV: 84.2% (79.5–88.1)
Total	141^a^	297	438	Positive LR: 187 (26.4–1324.8)
				Negative LR: 0.253 (0.195–0.320)

Sensitivity, specificity, positive and negative predicted values (PPV and NPV), positive and negative likehood ratios (LR) of SOFIA^®^RSV tested among infants, in the pediatric ED

*CI* Confidence Intervals

^a^19 SOFIA^®^RSV tests have been excluded since confirmation by RT-PCR has not been done for technical reasons

**Fig. 4:**
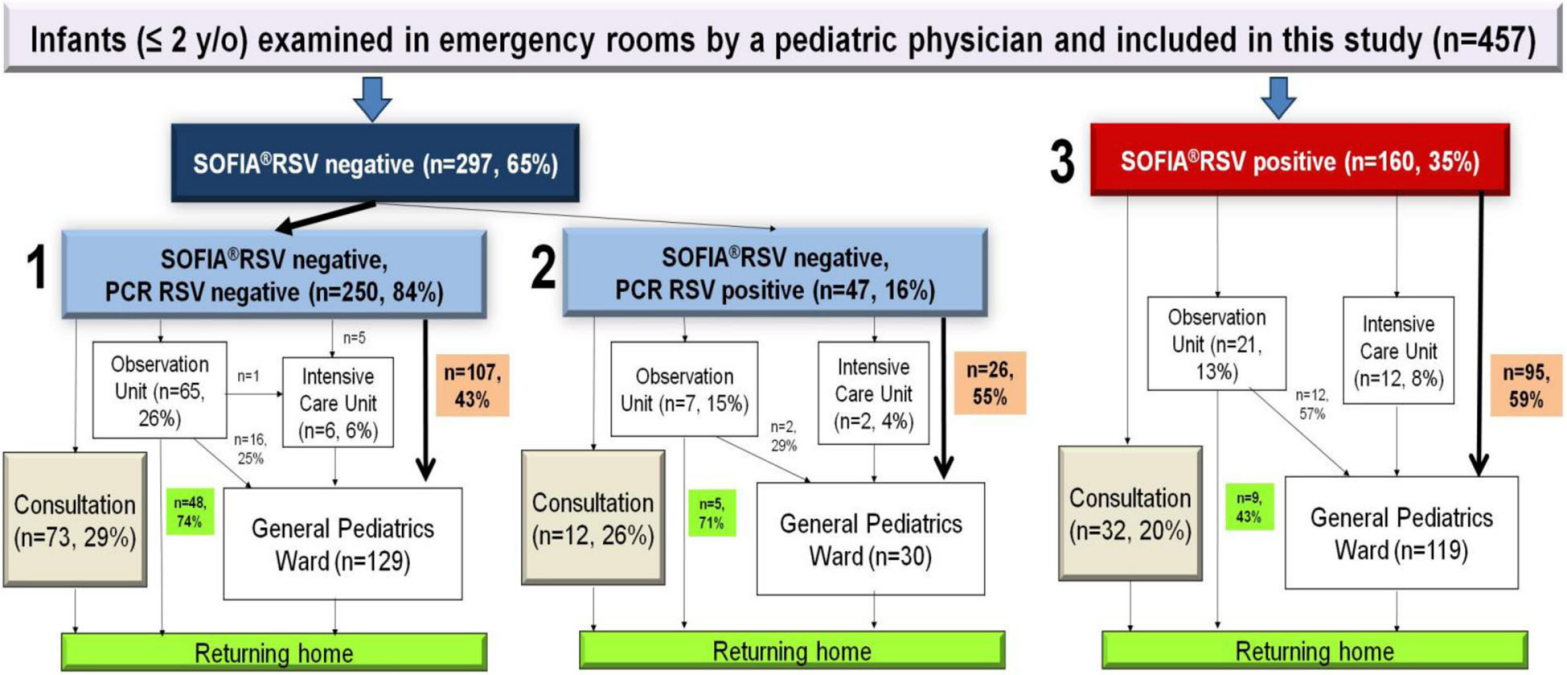
Study 2. Bed-management of infants SOFIA^®^RSV negative and SOFIA^®^RSV positive (SOFIA^®^RSV tests performed at POCT). Group 1: SOFIA^®^RSV negative and PCR RSV negative, Group 2: SOFIA^®^RSV negative and PCR RSV positive, Group 3: SOFIA^®^RSV positive

Three groups among infants were defined: group 1 including infants without RSV infection (*n* = 250); group 2 including infants with delayed diagnosis of RSV infection in the laboratory (*n* = 47), and group 3 including infants with RSV infection diagnosed by SOFIA^®^RSV at POCT (*n* = 160). The follow-up and bed-management of infants is described in Fig. 4.

In group 1 (RSV negative), 208 (83.2%) of the 250 RSV negative samples were positive for a virus other than RSV. Among these 208 samples, viruses other than RSV were codetected in 42 samples (2 viruses) and 2 samples (3 viruses). The three most frequently codetected viruses were hRV/EV (*n* = 181), hMPV (*n* = 44), and hCoV (*n* = 38). The median age was 5.5 months. In terms of bed-management, 73 (29.2%) were outpatients, 107 (42.8%) were hospitalized in the general pediatrics ward, 65 (26%) in observation unit (length of stay under 24 h), and 5 (2%) were transferred to PICU.

In group 2 (SOFIA^®^RSV negative and PCR RSV positive) of 47 samples, 27 (57.4%) were positive for at least one virus other than RSV. The three most frequently codetected viruses were human hRV/EV (*n* = 15), hCoV (*n* = 4), and HAdV (*n* = 3). The median age in group 2 was 3 months. They were 12 (25.5%) outpatients, 28 (55.3%) were hospitalized in the general pediatrics ward, 7 (14.8%) in the observation unit, and 4.3% (2/47) in the PICU.

In group 3 (SOFIA^®^RSV positive at POCT) of 160 samples, viral codetection was not attempted. The median age in group 3 was 3 months. In this group, 32 (20%) were outpatients, 95 (59.4%) were admitted to the general pediatrics ward, 21 (13.1%) to the observation unit, and 7.5% (12/160) to the PICU.

There were significant differences between groups 1–2 (SOFIA^®^RSV negative at POCT) and group 3 (SOFIA^®^RSV positive at POCT) with regard to admissions to the PICU (2% and 8% respectively, *P* < 0.001) and hospitalizations in the general pediatrics ward (41% and 59% respectively, *P* < 0.001) (Table 3). There were no significant differences in the bed-management of infants between groups 1 and 2, and between groups 2 and 3.

**Table 3:** Study 2 Statistical comparison in bed-management of patients having negative and positive SOFIA^®^RSV tests at point-of-care

	Groups 1 and 2 SOFIA^®^RSV negative (*n* = 297)	Group 3 SOFIA^®^RSV positive (*n* = 160)	*P*-value
Pediatric Emergency Department attendances (*n* = 117)	29% (*n* = 85)	20% (*n* = 32)	NS
Observation Unit (*n* = 93)	24% (*n* = 72)	13% (*n* = 21)	<0.01
General Pediatrics Ward (*n* = 228)	45% (*n* = 133)	59% (*n* = 95)	<0.01
Intensive care Unit (*n* = 19)	2% (*n* = 7)	8% (*n* = 12)	<0.05

Groups 1 and 2 are tested SOFIA^®^RSV negative, Group 3 are tested SOFIA^®^RSV positive. The number of patients in each unit refers to the number of patients who were directly hospitalized into this unit after having the SOFIA^®^RSV test result

*NS* Non significant. Chi^2^ test was used for statistical comparisons

Furthermore, we had a closer look at infants infected with RSV, whether the diagnosis was delayed (group 2) or directly made at POCT (group 3). Considering groups 2 and 3 together (*n* = 207), 163 (78.7%) have been hospitalized, among whom 135 (82.8%) stayed at least one day in the general pediatrics ward, with a median of stay of 4 days. Fourteen of these infants were hospitalized in the PICU. Of the 44 pediatric ED attendances from groups 2 and 3, 32 (72.7%) patients had tested SOFIA^®^RSV positive. These 32 patients were born at term (mean gestational age of 38 weeks), 24 (75%) showed signs of respiratory distress at initial examination and 27 (84.4%) were diagnosed with bronchiolitis.

Statistical comparisons in clinical presentation and prescribed treatment between groups 2 and 3 are detailed in Table 4. The statistically significant differences reflect early age of under 6 months (*P* < 0.05), signs of respiratory distress (*P* < 0.001), diagnosis of bronchiolitis (*P* < 0.001), and administration of an aerosol therapy, especially epinephrine aerosols (*P* < 0.05). There were no statistical differences between these two groups for the other clinical symptoms, for final diagnosis or for prescribed treatment using corticosteroids or antibiotics.

**Table 4:** Study 2. Comparison between two groups of infants (≤2y/o) diagnosed of RSV infection

	Group 2 Delayed RSV diagnosis at the laboratory (*n* = 47)	Group 3 RSV diagnosis at POCT (*n* = 160)	*P*-value	% of infants with RSV diagnosis
Clinical Symptoms				
Gestational age under 37 weeks	26% (*n* = 12)	14% (*n* = 22)	NS	16.43
Age under 6 months	72% (*n* = 34)	85% (*n* = 136)	<0.05	82.13
Temperature above 38 °C	36% (*n* = 17)	42% (*n* = 67)	NS	40.60
Signs of Respiratory Distress	68% (*n* = 32)	89% (*n* = 143)	<0.001	84.54
Feeding Difficulties	32% (*n* = 15)	33% (*n* = 52)	NS	32.37
Final diagnosis				
Bronchiolitis	64% (*n* = 30)	91% (*n* = 145)	<0.001	84.54
Pneumopathy	6% (*n* = 3)	9% (*n* = 15)	NS	8.71
Asthma	9% (*n* = 4)	3% (*n* = 5)	NS	4.35
Patients Management				
Corticosteroids	60% (*n* = 28)	72% (*n* = 115)	NS	69.08
Antibiotics	23% (*n* = 11)	20% (*n* = 32)	NS	20.77
Aerosol Therapy	72% (*n* = 34)	86% (*n* = 138)	<0.05	83.10
Epinephrine Aerosol	53% (*n* = 25)	71% (*n* = 113)	<0.05	

Infants with RSV diagnosis were compared at the laboratory (Group 2: SOFIA^®^RSV negative and PCR RSV positive) and at point-of-care testing (POCT, Group 3: SOFIA^®^RSV positive)

Aerosol Therapy included Epinephrine, Salbutamol, Ipratropium bromide, Budesonide or normal saline

*NS* Non Significant. Chi^2^ test was used for statistical comparison

Moreover, in the satisfaction surveys sent to 13 emergency physicians, the 9 responses received expressed satisfaction, indicating that using SOFIA^®^RSV tests had changed or improved their patient management.

## Discussion

In study 1, which was conducted in the laboratory, we showed that SOFIA^®^RSV is a sensitive and specific method for infants with high negative predictive value (96%), positive and negative likelihood ratios of 95 and 0.05, respectively. Its performance proves equivalent to PCR tests in infants. In the other age groups, the negative predictive values were similar to those calculated with the other direct detection techniques (DFA RSV and MRC5 RSV). This can be explained by the fact that among children younger than 2 y/o, viral replication during first infection is higher since they have no prior immunity [3]. Finally, study 1 is a prospective evaluation in a “real-life laboratory”, which provided the advantage of comparing the performance of several direct detection tests to PCR and allowed the calculation of positive, and especially negative predictive values. These data are often missing in recently published studies, where only values of sensitivities and specificities are shown [13–16].

Study 2 is an observation of the implementation, for the first time in France, of SOFIA^®^RSV at POCT in a pediatric ED. This RSV test is easy-to-use, including a printed report that describes the quality requirements for biological diagnosis. It is also an efficient method for making a quick and accurate diagnosis, which can save significant amount of time during a RSV epidemic period. Compared to PCR, this SOFIA^®^RSV test is faster, feasible at POCT, especially in smaller hospitals where no laboratory would be available and close to the pediatric ED. Diagnosing RSV at POCT is possible and highly informative. In fact, we showed significant differences in bed-management in infants: compared to SOFIA^®^RSV negative children, SOFIA^®^RSV positive children were more likely to be admitted to the PICU (*P* < 0.001) or to the general pediatrics ward (*P* < 0.001) (Table 3). There were also significant differences between infants with delayed RSV diagnosis (group 2) and with RSV diagnosis at POCT (group 3), concerning bronchiolitis diagnosis (*P* < 0.001) as well as aerosol therapy (*P* < 0.05) (Table 4). However, a treatment protocol has been implemented in the ED during the study 2 period, which recommended doctors to prescribe epinephrine aerosols to infants above 6 weeks of age who were diagnosed with bronchiolitis. Consequently, epinephrine aerosols cannot be taken into account in study 2, since the final diagnosis of bronchiolitis was particularly significant in group 3 (RSV infection diagnosed at POCT). In study 2, clinicians properly targeted the population at greatest risk of developing RSV disease (under 6 months of age, particularly under 3 months of age), in accordance with the national 2012 French guidelines which identify the target population during epidemics as infants under 4 months old [17]. There was no significant reduction in prescriptions for antibiotics or corticosteroids, whether the emergency physicians were aware of the presence of RSV or not, except for aerosols. These results may provide a rationale for clinical reflection, especially since the national guidelines [17] have highlighted the overuse of inappropriate (bronchodilators, corticosteroids) or deleterious (antitussive) medications from results of national surveys. These recommendations are also in the NICE 2015 guidelines [18]. That being said, this non-compliance with the guidelines, especially for antibiotic prescriptions, has been previously reported [19]. In Table 4, among the significant differences between groups 2 and 3, there were age under 6 months and the presence of signs of respiratory distress. These results suggest a viral load differential associated with whether the RSV diagnosis is delayed or made at POCT. Infants from group 3 had probably presented a higher viral load in the upper respiratory tract, consequently making the SOFIA^®^RSV more likely to be positive. Moreover, these patients presented initially with more severe symptoms at first examination. Previous studies showed that high viral load is a prognostic factor of severity [20, 21], since most children have apnea and lower weight at presentation. A young age has already proven to be a severity factor in RSV infections [22, 23]. We assume that RNA RSV load may have a positive correlation with longer hospitalizations and a greater use of intensive care.

The sensitivity of the SOFIA^®^RSV test in infants performed in study 1 conditions (collected in 1.5 mL of UTM) in the laboratory is 95% compared to 74.8% in study 2, in which tests were performed at POCT (collected in 3 mL of UTM). This higher volume of collection in study 2 could explain the decrease of sensitivity from the laboratory to the pediatric ED. Furthermore, we assume that what is not done in the laboratory can be associated with lower values of sensitivity. At POCT, the conditions are not those recommended by the manufacturer and could affect sensitivity. However, we chose to do differently for research purposes. Five studies from the United States [14, 15], Europe [16, 24] and Asia [25] have evaluated the SOFIA^®^RSV test performance between 2014 and 2015, in hospitals and clinics. Among them, 3 studies focused only on a pediatric population (under 18 y/o); the 2 other studies examined an adult population. No study has been conducted solely on infants, yet they form the most critical target population for RSV infection. Finally, only one study was conducted at POCT on a pediatric population, described by the mean and the median ages [24]. In that study, the SOFIA^®^RSV tests were performed on nasopharyngeal swabs placed in 1 mL of UTM, and showed a sensitivity of 81.8% compared to PCR in infants, whose proportion in the study population was not defined. Two other studies from Kanwar et al. [15] and Bruning et al. [16] showed sensitivity values equivalent to our results, for respiratory samples placed in 1 mL in the Kanwar study but not specified in the Bruning study. However, our results cannot be compared with these studies, since one was conducted in a laboratory and the other did not show the age distribution among the pediatric patients. The study of 348 respiratory samples reported by Jang et al. [25] was conducted on patients aged from birth to 98 y/o, with a mean age of 28 y/o, in South Korea. These data did not separate patients into age groups and therefore cannot be compared to our study, which is focused on infants. The study reported by Leonardi et al. [14] compared the performances of 4 rapid diagnostic tests with 230 samples placed in 3 mL of UTM, but the sampled population is also insufficiently described for comparison with our study. In 3 of the 5 studies, the samples were placed in 3 mL of UTM, similar to the modified protocol we used in study 2. Finally, our virological results are not outliers in terms of sensitivity: they are robust for infants, when nasopharyngeal swabs are placed in 1.5 mL of UTM. Despite the difficulties in comparing above mentioned studies, our results of sensitivity of 74.8% at POCT are similar to Bruning study (sensitivity of 75%, age non-specified). The robustness of the test guarantied accurate results even under poor sampling conditions.

The main limitation of study 2 was the retrospective clinical data collection, performed by different students, even though this collection was standardized with clear instructions. In contrast, study 1 was a prospective evaluation of SOFIA^®^RSV performance.

## Conclusions

Our work emphasizes the satisfactory performance level of SOFIA^®^RSV tests targeted in infants at POCT. Further reflection on the use of SOFIA^®^RSV tests in the pediatric ED would optimize patient management, when clinicians are globally satisfied. SOFIA^®^RSV tests will be very useful when new specific treatments become available, for example antiviral or immunomodulators, to reduce viral load and clinical severity scores. Further studies are needed to assess the economic impact of SOFIA^®^RSV within the national health system since RSV infections are associated with considerable costs [26].

## Additional file

Additional file 1: Study 2- SOFIA^®^RSV satisfaction survey. This survey was sent to the 13 pediatric consultants that were working in the ED during the study 2 period, 5 months after the end of the study. It includes 4 questions and one space for comments. (DOCX 11 kb)

## Abbreviations

ALRI: Acute lower respiratory infections
DFA: Direct fluorescent antibody
ED: Emergency department
FluA: Influenza virus type A
FluB: Influenza virus type B
hAdV: Human adenovirus
hCoV: Human coronaviruses
hMPV: Human metapneumovirus
hPIVs: Human parainfluenza viruses
hRV/EV: Human rhinovirus/enterovirus
Insee: Institut national de la statistique et des études économiques
NPV: Negative predictive value
PCR: Polymerase chain reaction
PICU: Pediatric intensive care unit
POCT: Point-of-care
PPV: Positive predictive value
RDT: Rapid diagnostic test
RSV: Respiratory syncytial virus
RT-PCR: Real-time polymerase chain reaction
UTM: Universal virological transport medium
