# Supplementary material for: SOFIA®RSV: prospective laboratory evaluation and implementation of a rapid diagnostic test in a pediatric emergency ward
Source: BMC Infect Dis. 2017 Jun 26;17:452. doi: 10.1186/s12879-017-2557-8 (PMC5485495; doi:10.1186/s12879-017-2557-8)
Supplement: Additional file 1: — Study 2- SOFIA®RSV satisfaction survey. This survey was sent to the 13 pediatric consultants that were working in the ED during the study 2 period, 5 months after the end of the study. It includes 4 questions and one space for comments. (DOCX 11 kb) [file 12879_2017_2557_MOESM1_ESM.docx]

Laboratory of Virology. CHU Caen. Pr. Astrid Vabret. Dr Léa Tran. September 25th, 2014

**EVALUATION of the SOFIA®VRS test**

**Pediatric Emergency Department, November 2013 – March 2014**

*Thank you for deleting as applicable.*

1) For a given patient, did the fact of knowing the test result (positive of negative) influence patient care?

- Positive test : YES NO

- Negative test: YES NO

If YES, please precise how:

- Did it help in your drugs prescription? (for example, stopping antibiotics)

- Did it reduce your prescriptions of additional exams? (for example, urine dipstick, procalcitonin, chest radiography)

- Did it help in making medical decision to discharge home, to hospitalize or to take isolation measures?

- Did it help in your discussion with parents?

2) Did you prescribe more virological tests while you were aware of its disponibility in the pediatric emergency department?

YES NO

3) What is your global satisfaction of the use of this test at point-of-care?

- Very satisfactory

- Satisfactory

- Moderately satisfactory

- A little bit satisfactory

- Not satisfactory

4) Will you agree with a second implementation of this test for the incoming RSV epidemic season?

YES NO

5) Comment :
